# Perspectives of clinical research on Shen-Shuai-Ning in the treatment of diabetic nephropathy

**DOI:** 10.3389/fmed.2024.1438266

**Published:** 2024-09-27

**Authors:** Na Gao, Xi-jun Zhang

**Affiliations:** Department of Nephrology, Yan’an People’s Hospital, Yan’an, Shaanxi, China

**Keywords:** diabetic nephropathy, Shen-Shuai-Ning, herbal medicine, treatment, perspective

## Abstract

Diabetic nephropathy (DN) is one of the most common chronic complications in patients with diabetes and remains a leading cause of end-stage renal disease. Despite current treatment strategies focusing primarily on blood glucose control and antihypertensive medications, their efficacy is often limited, failing to halt disease progression effectively. However, Shen-Shuai-Ning (SSN), a traditional Chinese medicine compound, has demonstrated significant clinical efficacy in DN treatment in recent years. SSN exerts multifaceted effects, including immunomodulation, attenuation of oxidative stress, and inhibition of fibrosis, leading to improvements in renal function indices and reductions in proteinuria levels, with favorable tolerability. Furthermore, when combined with conventional treatments, SSN exhibits enhanced therapeutic efficacy, providing a comprehensive and effective treatment strategy for DN patients. Future research endeavors should prioritize large-scale, multicenter clinical trials to validate its efficacy and safety across diverse populations, thereby further advancing its integration into clinical practice.

## 1 Introduction

### 1.1 Epidemiology of diabetic nephropathy

DN is one of the most common chronic complications in patients with diabetes and is a leading cause of end-stage renal disease. As the prevalence of diabetes continues to rise globally, the incidence of DN is also increasing. According to the International Diabetes Federation, in 2019, approximately 463 million people worldwide had diabetes, and 30–40% of these patients are expected to develop DN (1, 2). In China, over 120 million people have diabetes, making DN a primary cause of chronic kidney disease in the country (3).

### 1.2 The hazards and treatment challenges of DN

DN not only significantly reduces patients’ quality of life but also substantially increases healthcare burdens. Clinical features of DN include proteinuria, progressive decline in renal function, and hypertension, eventually leading to renal failure and a high incidence of cardiovascular diseases (4). Current treatment strategies for DN focus on blood glucose control, blood pressure management, and the use of renin-angiotensin system inhibitors, but their effectiveness is often limited, and they cannot halt disease progression (5). Additionally, existing treatments can have various adverse effects, further restricting their clinical use.

### 1.3 The potential of Shen-Shuai-Ning as a traditional Chinese medicine formula in treating DN

SSN is a compound formulation composed of several TCM herbs. Recently, it has shown promising clinical efficacy in treating DN. Its main components include Astragalus membranaceus, *Salvia miltiorrhiza*, and Rehmannia glutinosa, which have the functions of tonifying *Qi*, nourishing *Yin*, and promoting *Blood* circulation (6). Modern pharmacological studies have demonstrated that SSN exerts significant anti-inflammatory, antioxidant, and anti-fibrotic effects in the renal glomeruli (7). Numerous clinical studies have confirmed that SSN significantly improves renal function indices, reduces proteinuria, and enhances the quality of life in patients with DN (8). Compared to traditional treatment methods, SSN has fewer adverse effects, and its multi-target, multi-mechanism approach as a TCM compound helps comprehensively control the progression of DN.

Therefore, in-depth research into the mechanisms and clinical application prospects of SSN in treating DN is of great significance for improving treatment outcomes and reducing patient burdens. This study aims to systematically summarize the latest research progress on SSN in the treatment of DN, provide a scientific basis for clinical practice, and explore its future development directions.

## 2 Components and pharmacological mechanisms of SSN

### 2.1 Major components of SSN

SSN is a TCM formula comprising several key herbal ingredients. The primary components include Astragalus membranaceus (*Huang Qi*), *Salvia miltiorrhiza* (*Dan Shen*), and Rehmannia glutinosa (*Di Huang*). Each herb has a long history of use in TCM, known for distinct therapeutic properties (6).

### 2.2 Pharmacological actions of each component

Astragalus membranaceus is known for its immunomodulatory effects, enhancing immune function by stimulating macrophages and natural killer cells (9). Additionally, it possesses strong antioxidant properties, reducing oxidative stress by neutralizing free radicals (10). Astragalus also decreases inflammation by inhibiting pro-inflammatory cytokines (11).

*Salvia miltiorrhiza* improves microcirculation, enhancing blood flow and reducing blood stasis, which is beneficial for preventing microvascular complications in diabetes (12). Salvia contains active compounds such as salvianolic acids and tanshinones that offer potent antioxidant effects (13, 14). Furthermore, Salvia modulates inflammatory pathways, reducing inflammatory responses (13, 14).

Rehmannia glutinosa is traditionally used to nourish *Yin* and support kidney function, making it suitable for chronic kidney disease treatments (6, 11). It mitigates oxidative stress, protecting renal cells from damage (15), and inhibits renal fibrosis, a common pathological process in chronic kidney diseases, including DN (15).

### 2.3 Mechanisms of renal protection by SSN

SSN exhibits significant anti-inflammatory effects, which are critical in the progression of DN. The formula reduces levels of pro-inflammatory cytokines such as TNF-*α*, IL-1β, and IL-6, which are implicated in renal inflammation and damage (16, 17). Additionally, it inhibits the activation of NF-κB, a key regulator of inflammation, thereby reducing inflammatory responses in renal tissues (18).

Oxidative stress plays a significant role in the development of DN, and SSN enhances the body’s antioxidant defenses by upregulating the expression of antioxidant enzymes like superoxide dismutase and glutathione peroxidase (7). This enhancement reduces oxidative stress and protects renal cells from damage caused by high glucose levels and other metabolic disturbances (7, 18).

Renal fibrosis, especially tubulointerstitial fibrosis, is a hallmark of advanced DN. SSN helps reduce fibrosis by inhibiting the TGF-*β*/Smad signaling pathway, which is crucial in the development of renal fibrosis (19). This inhibition prevents the excessive deposition of extracellular matrix components such as collagen, thereby preserving the normal architecture and function of renal tissue (19, 20).

In summary, SSN’s multi-component and multi-target approach provides a comprehensive therapeutic strategy for DN. Its capabilities to modulate immune responses, reduce oxidative stress, and prevent fibrosis highlight its potential as a valuable treatment option for managing this complex condition.

## 3 Clinical research on SSN in treating DN

### 3.1 Overview of clinical research

SSN, a TCM formula, has shown promising efficacy in the treatment of DN. Numerous studies have evaluated its efficacy and safety, employing various research designs and methods, including randomized controlled trials, prospective and retrospective analyses (21–42) (Table 1).

**Table 1:** Clinical studies of Shen-Shuai-Ning in the treatment of diabetic nephropathy.

Study Ref	Publication type	Disease	Sample Size	Treatment	Main findings
Yan 2024 (21)	Randomized Controlled Trial	DN	120	SSN capsules and GLP-1RA	The combination of GLP-1RA and SSN capsules effectively improves metabolism, kidney function, and inflammation in DN patients, with notable efficacy and safety
Fang 2023 (22)	Retrospective study	DN	100	SSN and saxagliptin	SSN with saxagliptin effectively treats DN by improving kidney function, lowering blood sugar, reducing inflammation, and enhancing quality of life
Liang 2023 (23)	Randomized Controlled Trial	DN	86	SSN capsules and valsartan	SSN and valsartan effectively alleviates symptoms, improves kidney function, and reduces inflammation in DN patients
Ning 2023 (24)	Retrospective study	DN	70	SSN tablets and insulin glargine	Combining SSN tablets with insulin glargine improves renal function and lowers blood sugar in DN
Luo 2022 (25)	Randomized Controlled Trial	DN	80	SSN capsules and atorvastatin	Combining SSN with atorvastatin enhances kidney function and metabolic control in DN patients, reducing inflammation and oxidative stress without increasing adverse effects
Ding 2021 (26)	Retrospective study	DN	56	SSN and ferulic acid piperazine tablets	SSN and ferulic acid piperazine tablets improve inflammation, blood sugar, kidney function, and clinical outcomes in DN
Qiu 2021 (27)	Randomized Controlled Trial	DN	120	SSN capsules and repaglinide tablets	SSN capsules combined with repaglinide effectively treat DN by regulating blood sugar, reducing inflammation, improving kidney function, and ensuring safety
Gao 2020 (28)	Randomized Controlled Trial	DN	116	SSN granules and benazepril	SSN granules combined with benazepril can reduce blood sugar levels, oxidative stress, and inflammation, and improve kidney function in patients with DN
Qiu 2020 (29)	Randomized Controlled Trial	DN	104	SSN and canagliflozin tablets	SSN and canagliflozin tablets improve blood sugar control and reduce early kidney damage in DN patients with Qi and Yin deficiency and phlegm and stasis syndrome
Bai 2020 (30)	Randomized Controlled Trial	DN	150	SSN and valsartan	Valsartan and SSN for T2DM with hypertension effectively controls blood pressure and protects kidneys
Chen 2020 (31)	Randomized Controlled Trial	DN	65	SSN capsules and candesartan cilexetil	SSN and candesartan cilexetil effectively treat DN by improving kidney function, blood sugar levels, and oxidative stress, and reducing inflammation
Yao 2020 (32)	Randomized Controlled Trial	DN	60	SSN capsules	SSN capsules enhance treatment outcomes, mitigate micro-inflammation, and ameliorate clinical symptoms in DN and chronic renal failure
Yuan 2019 (33)	Randomized Controlled Trial	DN	110	SSN granules and valsartan	SSN granules and valsartan effectively improves early DN by reducing oxidative stress, inflammation, lowering blood sugar, and enhancing renal function
Huang 2019 (34)	Randomized Controlled Trial	DN	64	SSN tablets	SSN tablets and compound α-keto acid tablets shows significant efficacy in treating stage 5 DN in T2DM patients
Cui 2019 (35)	Randomized Controlled Trial	DN	92	SSN capsules and thioctic acid	Combining SSN capsules with thioctic acid injection effectively treats DN by improving renal function and regulating inflammatory factors with good safety
Wang 2017 (36)	Randomized Controlled Trial	DN	70	SSN granules and saxagliptin	SSN granules combined with saxagliptin tablets effectively treat DN, improving renal function, lowering blood sugar, and reducing inflammation
Wang 2017 (37)	Randomized Controlled Trial	DN	60	SSN capsules	SSN capsules demonstrate significant clinical efficacy in treating DN
Deng 2016 (38)	Randomized Controlled Trial	DN	88	SSN and captopril	SSN and captopril effectively suppresses inflammatory response, delays renal damage, and improves therapeutic efficacy in the treatment of DN
Li 2015 (39)	Randomized Controlled Trial	DN	204	SSN granules and telmisartan tablets	SSN granules and telmisartan tablets reduce urinary albumin while stabilizing serum creatinine
Su 2010 (40)	Randomized Controlled Trial	DN	198	SSN and pancreatic kininogenase	Combining kininogenase with SSN effectively delays renal function decline in early-stage diabetic nephropathy, postponing the need for hemodialysis initiation
Lu 2009 (41)	Randomized Controlled Trial	DN	60	SSN capsules	SSN capsules effectively delay the progression of renal dysfunction in both compensated and decompensated stages of DN
Zhang 2002 (42)	Randomized Controlled Trial	DN	98	SSN	SSN capsule significantly improves renal function and alleviates clinical symptoms in DN with renal failure, achieving a better total effective rate

DN, diabetic nephropathy; SSN, Shen Shuai Ning; T2DM, type 2 diabctes mellitus.

### 3.2 Research design and methods

Several studies utilized random number tables for patient allocation to ensure randomness and reliability (21, 23, 27). Retrospective studies selected patients based on medical records, grouping and analyzing those meeting the criteria (22, 26). Study durations varied from several months to several years, covering different stages and types of DN patients (Table 1).

### 3.3 Patient selection and grouping

Studies typically selected DN patients meeting specific diagnostic criteria, such as particular renal function indicators and urinary protein levels. Patients were randomly assigned to treatment and control groups, with the treatment group receiving SSN combined with standard therapy, while the control group received only standard therapy or SSN alone (21, 22, 26) (Table 1). Selection criteria generally included age, gender, disease duration, and underlying conditions to ensure the homogeneity and comparability of study subjects (24, 25) (Table 1).

### 3.4 Treatment protocols and dosages

Treatment protocols included different formulations of SSN (such as capsules, tablets, granules) and dosages, and whether it was combined with other medications (e.g., valsartan, telmisartan) (25, 32, 39) (Table 1). Dosages and treatment durations were adjusted based on disease severity and specific study goals. For instance, some studies used SSN capsules three times daily, four capsules each time, combined with other medications for 2 to 3 months (37, 38) (Table 1).

### 3.5 Main study results

#### 3.5.1 Improvement in renal function

Most studies reported significant improvements in renal function indicators such as serum creatinine, blood urea nitrogen, and urinary protein excretion rate (34, 35, 37) (Table 1). For example, one study showed that serum creatinine and blood urea nitrogen levels significantly decreased in the treatment group after therapy, and were lower than those in the control group, indicating SSN’s efficacy in enhancing renal function (36) (Table 1).

#### 3.5.2 Changes in urinary protein excretion

Urinary protein excretion is a key indicator for assessing DN treatment efficacy (Table 1). Studies have shown that SSN effectively reduces urinary protein excretion, slowing the progression of DN (28, 36). For instance, in one study, the 24-h urinary protein excretion significantly decreased in the treatment group and was lower than in the control group, demonstrating SSN’s protective effect on the kidneys (33).

#### 3.5.3 Changes in blood glucose and other metabolic indicators

SSN also has a positive effect on blood glucose control in diabetic patients, reducing levels of glycated hemoglobin and fasting blood glucose (26, 30) (Table 1). Additionally, it has been observed to positively impact other metabolic indicators such as blood lipids (Table 1). For example, studies have shown that total cholesterol and low-density lipoprotein levels significantly decreased, while high-density lipoprotein levels increased in patients treated with SSN (25).

### 3.6 Safety and adverse reactions

#### 3.6.1 Common adverse reactions and management

Reports of adverse reactions to SSN are relatively few and mostly mild to moderate, including gastrointestinal discomfort (33, 38) (Table 1). These reactions are typically self-resolving or can be managed by adjusting the dosage and treatment regimen. Reports of severe adverse reactions are rare, with no significant life-threatening or serious harm events observed (29, 40).

#### 3.6.2 Safety evaluation

Based on multiple studies, SSN has demonstrated a high safety profile in treating DN. Most studies did not find significant serious adverse reactions (31, 41) (Table 1). During treatment, patients’ renal function and other biochemical indicators significantly improved without noticeable adverse reactions, indicating its high clinical application value and safety (32, 42) (Table 1).

## 4 Mechanisms of SSN in treating DN

Understanding the mechanisms underlying SSN’s therapeutic effects in DN entails a comprehensive exploration of its actions at various levels, spanning cellular, animal model, and molecular biology research (6, 16–20). This investigation elucidates how SSN attenuates the progression of DN and offers insights into its clinical application.

### 4.1 Cellular-level research

#### 4.1.1 Interactions between glomerular and tubular cells

At the cellular level, SSN intervenes in DN by targeting both glomerular and tubular cells within the kidney. These cells play pivotal roles in the pathogenesis of renal damage associated with diabetes (6, 43). SSN’s multi-herbal formulation exhibits protective effects by ameliorating glomerular dysfunction, including podocyte injury and mesangial cell proliferation, while also mitigating tubular cell injury, reducing tubular epithelial cell apoptosis, and promoting tubular cell regeneration (18). By preserving the structural and functional integrity of glomerular and tubular cells, SSN effectively impedes the progression of renal dysfunction in DN.

### 4.2 Animal model studies

#### 4.2.1 Mechanisms in DN animal models

Animal model studies serve as crucial platforms for elucidating the mechanisms underlying SSN’s therapeutic efficacy in DN. Utilizing various models, such as streptozotocin-induced diabetic rats and high-fat diet-induced diabetic mice, researchers have demonstrated SSN’s significant renal protective effects (18). These effects encompass attenuation of renal hypertrophy, reduction in urinary albumin excretion, and amelioration of renal histopathological alterations. Mechanistically, SSN intervenes by suppressing inflammatory responses, ameliorating oxidative stress, and inhibiting renal fibrosis in diabetic animals (43). These findings underscore the multifaceted nature of SSN’s therapeutic action, which involves modulation of diverse pathogenic pathways implicated in DN.

### 4.3 Molecular biology research

#### 4.3.1 Signaling pathways and gene expression)

Molecular biology investigations provide valuable insights into the signaling pathways underpinning SSN’s therapeutic effects in DN. By targeting key pathways such as the TGF-*β*/Smad pathway, and NF-κB pathway, SSN orchestrates a comprehensive array of molecular responses (18). It modulates the expression of genes involved in inflammation, oxidative stress, apoptosis, and fibrosis, thereby exerting profound renal protective effects (43). Moreover, the synergistic interactions among SSN’s constituent herbs contribute to its enhanced therapeutic efficacy compared to single-component interventions.

In summary, SSN’s therapeutic mechanisms in DN encompass a multifaceted approach, involving interventions at the cellular, animal model, and molecular levels. By targeting glomerular and tubular cells, suppressing inflammation, ameliorating oxidative stress, and inhibiting renal fibrosis, SSN offers a holistic therapeutic strategy for managing DN. Further elucidation of its mechanisms holds promise for the development of innovative therapeutic interventions aimed at combating this debilitating condition.

## 5 Comparison of SSN with existing treatment methods

### 5.1 Conventional medicine treatment regimens

The primary treatment options for DN in Western medicine include Angiotensin-Converting Enzyme Inhibitors (ACEIs) or Angiotensin II Receptor Blockers (ARBs) and statins. These medications are widely used due to their proven efficacy in managing DN.

#### 5.1.1 ACEIs/ARBs

ACEIs and ARBs are fundamental in managing DN as they reduce proteinuria and slow kidney disease progression by controlling blood pressure and reducing intraglomerular pressure. Studies indicate these medications significantly decrease the risk of renal failure and delay progression to end-stage renal disease (30, 33). Common ACEIs include enalapril and lisinopril, while losartan and valsartan are frequently prescribed ARBs.

#### 5.1.2 Statins

Statins are primarily prescribed for managing hyperlipidemia, which is common in diabetic patients, and they offer cardiovascular protection. These medications work by inhibiting HMG-CoA reductase, an enzyme crucial for cholesterol synthesis. Statins like atorvastatin and rosuvastatin reduce low-density lipoprotein cholesterol and total cholesterol, beneficial in mitigating oxidative stress and inflammation associated with DN (25).

### 5.2 Effects of combined treatment with SSN

Combining SSN with conventional Western medications has demonstrated enhanced therapeutic outcomes in DN patients, providing synergistic benefits.

#### 5.2.1 Synergistic effects with Western medications

The combination of SSN with ACEIs or ARBs has shown superior renal protection compared to Western medications alone. Research has demonstrated that combined therapy significantly reduces serum creatinine and blood urea nitrogen levels more effectively than ACEIs or ARBs alone (21, 31). For instance, a study found that combining SSN with valsartan led to greater improvements in renal function and a more substantial reduction in urinary protein excretion than valsartan alone (30). This synergy is likely due to SSN’s ability to enhance blood circulation and reduce inflammation, complementing the renal protective effects of ACEIs and ARBs.

Similarly, combining SSN with statins results in better control of lipid profiles and reduced oxidative stress markers. The antioxidant properties of SSN, combined with the lipid-lowering effects of statins, provide a comprehensive approach to managing DN. Studies have reported that patients receiving this combination therapy showed significant improvements in both renal function and lipid metabolism compared to those receiving only statins (25, 35).

#### 5.2.2 Effects of combination with other Chinese medicines

SSN has also been studied in combination with other TCM, showing promising results. For instance, combining SSN with Ferulic Acid Piperazine has been shown to effectively reduce inflammation and improve renal function markers such as serum creatinine and urinary protein levels (26). Another study highlighted the benefits of combining SSN with other herbal formulations like Astragalus, demonstrating enhanced therapeutic effects in reducing proteinuria and improving overall kidney function (37).

These combinations leverage the multi-targeted approach of TCM, which focus on restoring systemic balance and improving organ function through various mechanisms. This holistic approach often results in better patient outcomes and improved quality of life, as evidenced by several clinical studies (32, 38).

SSN, both as a monotherapy and in combination with conventional Western medicines, offers significant advantages in managing DN. Its integration with ACEIs, ARBs, and statins enhances therapeutic effects, leading to improved renal function and better metabolic control. Additionally, its combination with other TCM provides a comprehensive treatment approach for DN, highlighting its potential as a valuable component of integrated treatment regimens.

## 6 Prospects for SSN in the treatment of DN

### 6.1 Future research directions

#### 6.1.1 Large-scale, multicenter clinical trials

Future research on SSN should prioritize large-scale, multicenter clinical trials to thoroughly validate its efficacy and safety (44). These trials would provide robust data across diverse populations and healthcare settings, addressing the limitations of smaller, single-center studies (44). By involving a larger sample size and multiple centers, the generalizability of the results can be enhanced, offering a more accurate assessment of SSN’s therapeutic potential in treating DN.

#### 6.1.2 Long-term efficacy and safety studies

Investigating the long-term efficacy and safety of SSN is crucial. While short-term studies have shown promising results, understanding the long-term effects is essential. This includes evaluating the sustainability of benefits on renal function, glucose metabolism, and overall patient health, as well as monitoring for any long-term adverse effects. Long-term studies would provide valuable insights into the chronic management of DN and the role of SSN, ensuring it remains a safe and effective treatment option over extended periods.

### 6.2 Challenges in clinical application

#### 6.2.1 Standardization and regulation

A significant challenge in the clinical application of SSN is the standardization and regulation of its production and use. TCM often faces issues related to batch-to-batch consistency and quality control due to variations in raw materials and preparation methods (45, 46). Ensuring that SSN is produced under stringent quality control measures and adheres to standardized protocols is crucial for its widespread clinical adoption (45, 46). Establishing and enforcing regulatory frameworks will ensure the safety, efficacy, and quality of SSN, thereby building trust among healthcare providers and patients.

#### 6.2.2 Implementation of personalized treatment

Achieving personalized treatment with SSN presents another challenge. DN patients exhibit significant variability in disease progression, treatment response, and underlying health conditions. Therefore, a one-size-fits-all approach may not be effective. Future research should focus on identifying biomarkers and patient characteristics that predict response to SSN, enabling the development of tailored treatment regimens (47). Personalized medicine approaches, including pharmacogenomics and patient stratification based on genetic, biochemical, and clinical profiles, can optimize treatment outcomes and minimize adverse effects (48).

SSN holds significant promise in treating DN, but its future success depends on overcoming current challenges through rigorous research and standardization. Large-scale, multicenter trials and long-term studies are necessary to establish its efficacy and safety comprehensively. Addressing standardization issues and advancing personalized treatment approaches will be crucial for its effective integration into clinical practice. With these efforts, SSN can potentially become a cornerstone in the holistic management of DN, combining the strengths of TCM with modern medical practices.

## 7 Summary

### 7.1 Summary of research progress

SSN has demonstrated significant potential in treating DN, with clinical studies showing improvements in renal function, reduced urinary protein excretion, and enhanced metabolic control. Combining SSN with conventional treatments like ACEIs, ARBs, and statins has provided synergistic benefits, leading to better clinical outcomes. Its favorable safety profile, with few reported adverse effects, supports its use as a therapeutic option for DN.

### 7.2 Outlook for future research and clinical application

Future research should focus on large-scale, multicenter clinical trials to validate SSN’s efficacy and safety across diverse populations. Long-term studies are needed to assess the sustainability of its benefits and monitor for chronic adverse effects. Standardizing production and regulatory practices will ensure consistency and quality, fostering greater trust among healthcare providers and patients. Advancing personalized treatment approaches by identifying biomarkers that predict response to SSN will optimize outcomes and minimize adverse effects. Overcoming these challenges will enable SSN to become a cornerstone in managing DN, integrating TCM with modern healthcare practices.

## Data Availability

The original contributions presented in the study are included in the article/supplementary material, further inquiries can be directed to the corresponding author.
